# Environmental impact of the cultivation of energy willow in Poland

**DOI:** 10.1038/s41598-021-84120-0

**Published:** 2021-02-25

**Authors:** Zbigniew Kowalczyk, Dariusz Kwaśniewski

**Affiliations:** Faculty of Production Engineering and Energetics, University of Agriculture in Krakow, ul. Balicka 116B, 30-149, Kraków, Poland

**Keywords:** Ecology, Environmental sciences

## Abstract

The purpose of the work is to analyze the structure of the environmental impact of energy willow cultivation (*Salix* spp*.*) on plantations of various sizes, divided per materials and processes. The research covered 15 willow plantations, ranging from 0.31 ha to 12 ha, located in southern Poland. It was found, among others, that the so-called processes, i.e. the use of technical means of production, dominate the structure of the environmental impact (EI) related to the cultivation of energy willow, and that the cultivation of energy willow on larger plantations has a much lower environmental impact compared to cultivation on smaller plantations. Also, in the case of the environmental impact of processes, the largest environmental impact was recorded in the human health category, which is mainly associated with the consumption of fuel, i.e. diesel. It was determined, e.g., that the cultivation of energetic willow on larger plantations is characterized by a much lower environmental impact (as per the cultivation area), at approx. 108 Pt, compared to the cultivation on smaller plantations, where the value of the environmental impact is 168 Pt. A decisively dominant position in the structure of the environmental impact (EI), related to the cultivation of energy willow, is held by the so-called processes, i.e. the use of technical means of production. Their share in the total environmental impact decreases from 148.5 Pt in the group of the smallest plantations to 77.9 Pt in the group of the largest plantations.

## Introduction

Growing concerns about climate change, geopolitical uncertainty associated with continuous energy supply, and increasing costs of fossil fuels motivate the search for clean and renewable conventional fuel substitutes^1–3^. One alternative to fossil fuels is the so-called bioenergy, the interest in which has increased significantly in recent years^4^. Bioenergy can be obtained from biomass used for the production of fuels, which in turn can be processed to obtain power, heat, and transport fuels^5,6^. As a source of renewable energy, biomass has enjoyed great interest for some time, also due to the postulates of the so-called sustainable energy management and the reduction of greenhouse gas emissions. The European Union has adopted several goals for the year 2020, e.g.: 20% reduction in greenhouse gas emissions, a 20% increase in energy efficiency, and reaching a 20% share of renewable energy in total energy produced^7^. Biomass for bioproducts and bioenergy can be obtained from forests, arable crops, various waste, and dedicated wood or herbaceous crops^8,9^. Work is currently underway on short rotation woody crops (SRWC) to provide a renewable raw material for bioenergy production^10^. SRWC, such as willow (*Salix* spp.), and poplar, are an important source of renewable energy and can be converted into electric power and/or heat using conventional or modern biomass processing technologies^11^.

SRWC is a fast-growing, high-yield energy plant that can not only be burned but also gasified to generate heat and energy^11,12^. There are about 450 varieties of willow globally^13^. This perennial energy plant is known for its high biomass yield in a short time and a broad genetic pool^14^. Moreover, willow shrubs are characterized by easy vegetative reproduction from dormant hardwood cuttings, easy cultivation, and the ability to regenerate after many harvests^15,16^. Its advantage is also that willow can be grown on arable land^17^.

According to Heller^18^, the production of biomass from willow plants requires the use of 0.018 MJ of non-renewable energy to produce 1 MJ of renewable energy in the form of wood fuel. In turn, the production of power from dedicated energy willow crops, 0.092 MJ of non-renewable energy is consumed per 1 MJ of generated power^18^. Many species of willow are a promising source of woody biomass, which can be very beneficial for the development of rural areas, apart from the environmental aspects^19,20^. Compared to annual cultivation systems, perennials, including willow are less susceptible to adverse weather conditions or epidemics of pests and diseases when it comes to crop yields^21,22^. The transformation of land into perennial bioenergy crops promotes the increase of some types of habitats of wild fauna and flora and reduces GHG emissions, depending on the nature and location of the plantation^20,23^. Moreover, it can contribute to soil organic carbon storage^24^ and in the short term, alleviate the effects of high CO2 concentrations^25^. Perennial willow plantations can effectively absorb groundwater and soil pollution and be used in land reclamation^26^. Agricultural biomass may be of interest for the purposes of energy production, especially in rural areas where end-users are in the vicinity of biomass farms. This would avoid problems with the transport of biomass, which generally has a low bulk density and low energy value in the unconcentrated state^27^. However, many researchers point out that, unfortunately, large-scale conversion of land to bioenergy production may have serious ecological effects^28–31^ because it affects wildlife habitats^32^ and competes for arable land with food crops^33^. The use of agricultural land for the production of raw material energy crops can have an indirect negative environmental effect. This is due to a significant intensification or expansion of agricultural production elsewhere to compensate for the lost food production^34^. Land conversion can also disrupt ecosystem cycles such as the hydrolog^35^, soil storage of organic carbon^36–40^, and nutritional cycles^41,42^. Potentially, changes in land use can also increase GHG emissions^43–45^. Due to some unfavorable aspects of the use of biomass, opinions are also voiced that the production of energy crops should be minimized and the focus should be on the available, yet unused biomass resources^46^. After all, the use of biomass as an energy source can cause emissions that are harmful to human health and the environment, both on a local and global scale^47^.

In recent years, the life cycle assessment (LCA), which is increasingly used to assess the potential environmental impact of production systems^48–50^, including the assessment of the environmental impact of energy crops, has enjoyed considerable interest^22,51–53^. In LCA, the potential environmental impacts associated with the product/service life cycle are assessed based on the life cycle inventory (LCI), which includes the relevant input and output data, as well as emissions included in the system associated with the product/service^54^. LCA goals can include (1) comparison of alternative products, processes, or services; (2) comparison of alternative life cycles for a specific product or service; (3) identifying parts of the life cycle in which major improvements can be made^1^. Taking into account the entire life cycle, the life cycle assessment (LCA) reduces the risk of transferring problems from one production phase to another^55^ and is based on ISO 14,040 and ISO 14,044 standards. Unfortunately, the knowledge of the life cycle of energy willow biomass production is still limited^10,56^. Literature resources offer studies on the use of the LCA methodology in renewable energy production, but in the case of energy willow, unfortunately, the amount of the studies is relatively small. In many cases, the available research results were published many years ago, which depreciates them due to technological progress. Moreover, there are very few up-to-date results regarding the environmental impact of the very process of willow cultivation, and the published research results very often relate to the processes of converting willow biomass into various forms of energy (power, liquid fuels, heat). Moreover, the methodology used in individual studies of various authors is very different in terms of system boundaries and methods used, which means that they are not always comparable.

Comparing the positive and negative aspects of biomass utilization, there is a need for a comprehensive assessment of the impact of both the technologies related to the energy willow biomass production cycle and those related to processing technology as an energy source.

The purpose of the work was to analyze the structure of the environmental impact of the cultivation of energy willow on plantations of various sizes, divided per production materials and processes (Table 1).

**Table 1:** Abbreviations/nomenclatures.

CFC-11	Chlorofluorocarbon-11
1,4-DB	1,4 dichlorobenzene
NMVOC	Non-methane volatile organic compounds
PM10	Particulate matter formation
kBq U235	Equivalent uranium radiation measured in kilo Becquerel
EI	Environmental impact
Pt	One thousandth of the yearly environmental load of one average European inhabitant

## Materials and methods

### Research subject

The amounts of materials and energy included in each process have been calculated based on research carried out on energy willow farms. The research consisted of a detailed analysis of technologies related to willow cultivation. The research covered^15^ energy willow plantations located in southern Poland. The selection of willow plantations was deliberate, i.e. five plantations with a relatively small area (0.48 ha on average) were selected as Group I, five larger, with an average area of 2.08 ha, were included in Group II and five largest (average area—8.06 ha)—in Group III.

General information on energy willow plantations selected for testing is presented in Table 2. The yield of fresh biomass cut after the first year of cultivation ranged from 6.8 t·ha-1 (in Group I) to 9.4 t·ha-1 (in Group II). Energy willow plantations were located relatively close to the farm (from 0.6 km to 1.4 km on average), which impacted the scope of transportation works.

**Table 2:** General information regarding the plantations included in the study^57^.

Area group	Area min–max (average) (ha)	Number of plantations	Biomass yield (t·ha^−1^)	Distance min–max (average) (km)
I	0.31–1.00 (0.48)	5	5.9–7.2 (6.8)	0.6–1.6 (1.2)
II	1.20–4.00 (2.08)	5	8.1–10.2 (9.4)	0.9–1.7 (1.4)
III	5.19–12.00 (8.06)	5	7.2–8.7 (7.8)	0.4–1.1 (0.8)

**Figure 1:**
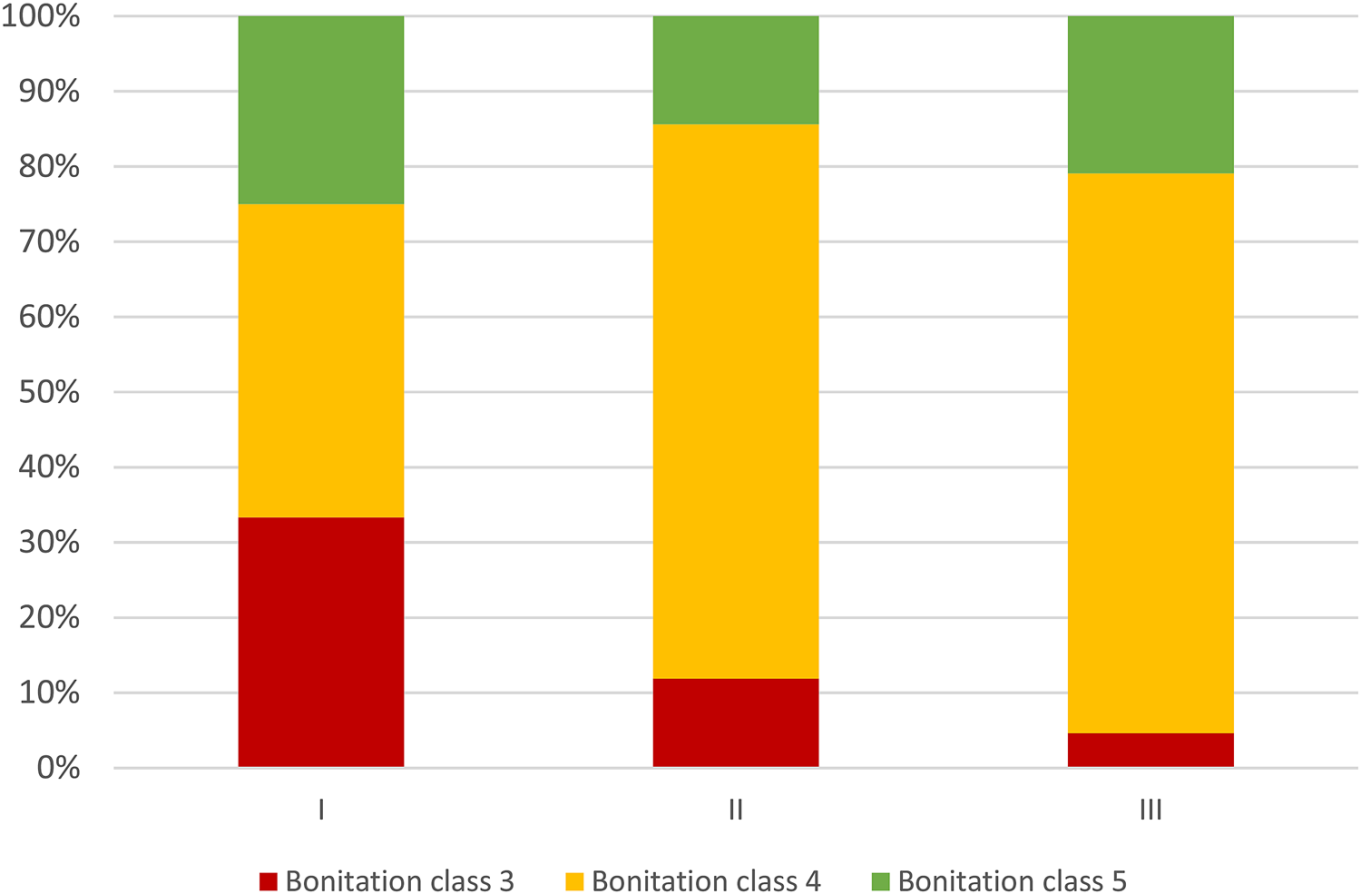
The structure of soil quality (bonitation) in particular area groups.

Upon analyzing Fig. 1, it can be observed that willow plantations were located in areas with rather low soil quality. In Group I, 66% of the total plantation area was located on soils of the 4th and 5th class, and in Group III, up to 95%.

### System boundaries

**Figure 2:**
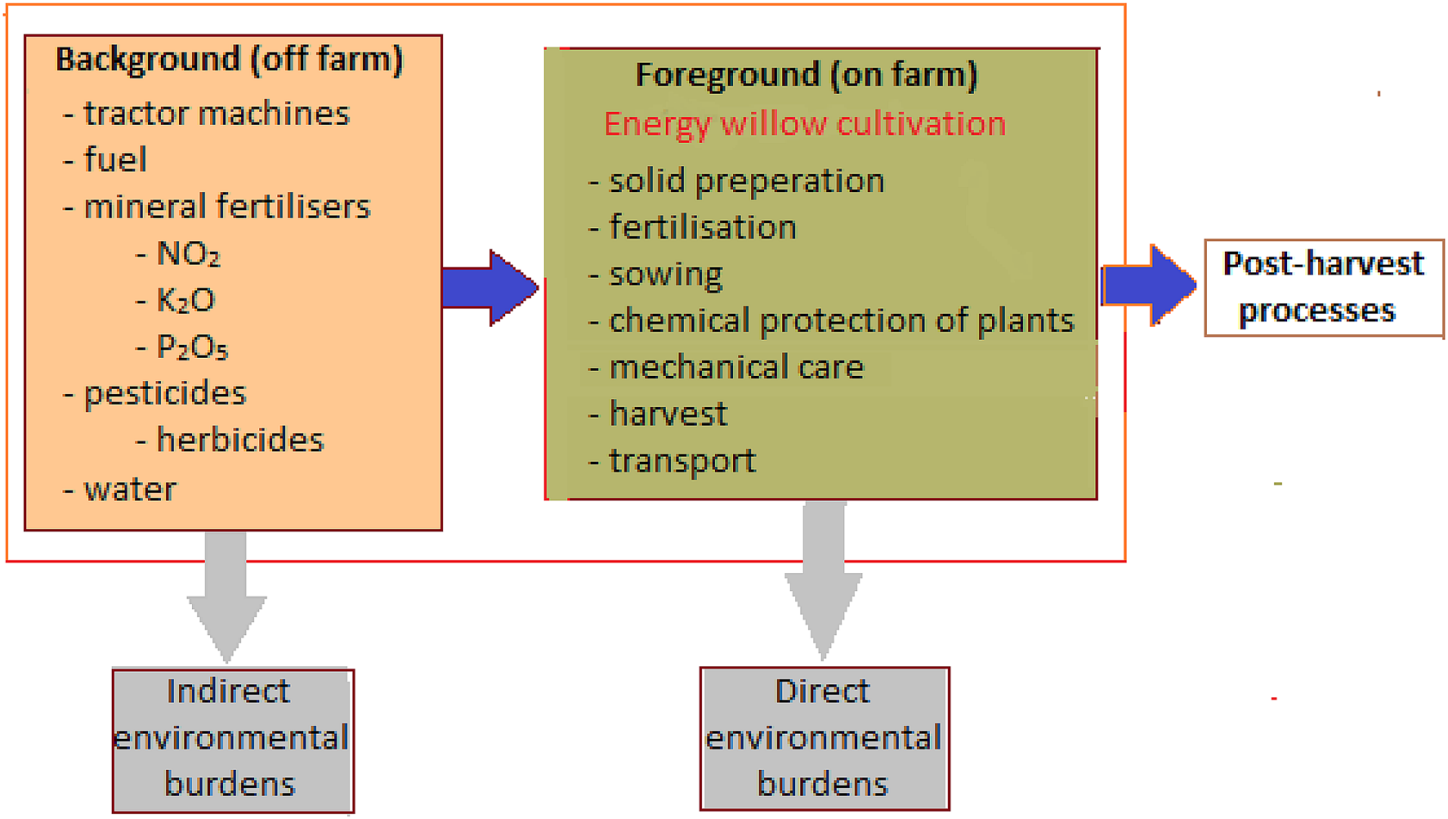
System boundaries.

The system boundaries are shown in Fig. 2. The analysis covered only activities related to the establishment of the plantation, its cultivation for one year, as well as the collection and transport of cut biomass. The willow seedlings used were not included in the analysis, due to the lack of such an item in the catalogs of SimaPro software. Post-harvest operations related to the harvested biomass were also omitted. The analysis also excluded the transport of fuels, fertilizers, and pesticides from purchase points to the farm.

### Impact assessment methodology

To assess the environmental impact of the cultivation of energy willow, the Life Cycle Assessment (LCA) method was used to determine the environmental relationships of all entrances and exits within the scope of the study, and to estimate the magnitude of their impact on the environment. The LCA method was applied using the SimaPro software, version 8.1.0.60. The detailed environmental impact assessment methods, ReCiPe Endpoint (H) V1.12/Europe ReCiPe H/A and ReCiPe Midpoint (H) V1.12/Europe Recipe H were used in the detailed calculations. The midpoint impact categories, the related indicators, and the key references were presented in Table 3. Endpoint indicators determine the environmental impact at three levels of aggregation, namely: (1) effect on human health (2) biodiversity, ecosystem and (3) resource scarcity. Environmental impact (EI) was calculated in the so-called units of general nuisance (Pt), used quite commonly in the LCA methodology, and related to one ton of fresh biomass yield and the cultivation area. The environmental impact of the use of production materials (materials) and the environmental impact of the use of technical means of production (processes) was estimated separately. The applied LCA methodology is based on the ISO 14,040 standard. The toxicity potential (TP), expressed in kg 1,4-dichlorobenzeneequivalents (1,4DCB-eq), is used as a characterization factor at the midpoint level for human toxicity, freshwater aquatic ecotoxicity, marine ecotoxicity and terrestrial ecotoxicity^73^.

**Table 3:** Overview of the midpoint impact categories and related indicators^58^.

Midpoint impact category	Indicator	CF_m_	Unit	Key references
Climate change	Infrared radiative forcing increase	Global warming potential (GWP)	kg C02-eq to air	IPCC^59^ Joos et al.^60^
Ozone depletion	Stratospheric ozone decrease	Ozone depletion potential (ODP)	kg CFC-11-eq to air	WMO 2011^61^
Ionising radiation	Absorbed dose increase	Ionising radiation potential (IRP)	kBq Co-60-eq to air	Frischknecht et al.^62^
Fine particulate matter formation	PM2.5 population intake increase	Particulate matter formation potential (PMFP)	kg PM2.5-eq to air	Van Zelm et al.^63^
Photochemical oxidant formation: terrestrial ecosystems	Tropospheric ozone increase	Photochemical oxidant formation potential: ecosystems (EOFP)	kg NOx-eq to air	Van Zelm et al.^63^
Photochemical oxidant formation: human health	Tropospheric ozone population intake increase	Photochemical oxidant formation potential: humans (HOFP)	kg NOx-eq to air	Van Zelm et al.^63^
Terrestrial acidification	Proton increase in natural soils	Terrestrial acidification potential (TAP)	kg S02-eq to air	Roy et al.^64^
Freshwater eutrophication	Phosphorus increase in freshwater	Freshwater eutrophication potential (FEP)	kg P-eq to freshwater	Helmes et al.^65^
Human toxicity: cancer	Risk increase of cancer disease incidence	Human toxicity potential (HTPc)	kg 1,4-DCB-eq to urban air	Van Zelm et al.^66^
Human toxicity: non-cancer	Risk increase of non-cancer disease incidence	Human toxicity potential (HTPnc)	kg 1,4-DCB-eq to urban air	Van Zelm et al.^66^
Terrestrial ecotoxicity	Hazard-weighted increase in natural soils	Terrestrial ecotoxicity potential (TETP)	kg 1,4-DCB-eq to industrial soil	Van Zelm et al.^66^
Freshwater ecotoxicity	Hazard-weighted increase in freshwaters	Freshwater ecotoxicity potential (FETP)	kg 1,4-DCB-eq to freshwater	Van Zelm et al.^66^
Marine ecotoxicity	Hazard-weighted increase in marine water	Marine ecotoxicity potential (METP)	kg 1,4-DCB-eq to marine water	Van Zelm et al.^66^
Land use	Occupation and time-integrated land transf	Agricultural land occupation potential (LOP)	m2 × year annual cropland-eq	De Baan et al.^67^ Curran et al.^68^
Water use	Increase of water consumed	Water consumption potential (WCP)	m3 water-eq consumed	Doll and Siebert^69^ Hoekstra and Mekonnen^70^
Mineral resource scarcity	Increase of ore extracted	Surplus ore potential (SOP)	kg Cu-eq	Vieira et al.^71^
Fossil resource scarcity	Upper heating value	Fossil fuel potential (FFP)	kg oil-eq	Jungbluth and Frischknecht^72^

### Life cycle inventory (LCI)

Table 4 summarizes the means of production used in willow cultivation technologies for each of the groups. The amounts of the so-called pure fertilizer component expressed as N, P_2_O_5_, and K_2_, prove that mineral fertilizers were relatively rarely used and only in selected plantations of Groups II and III. In turn, the largest consumption of pesticides (3.42 kg·ha^-1^ on average) was recorded in the group of the smallest plantations. The used pesticide was mainly the herbicide Roundup, applied before planting to destroy weeds. The willow plantations were not artificially irrigated, and the water consumption presented in Table 4 was only associated with performing chemical plant protection treatments.

**Table 4:** Selected means of production used in the cultivation of energy willow in individual groups^57^.

Specification	Group I		Group II		Group III	
	(kg·ha^−1^)	(kg·t^−1^)	(kg·ha^−1^)	(kg·t^−1^)	(kg·ha^−1^)	(kg·t^−1^)
Mineral fertilizers						
N	0.00	0.00	2.77	0.29	0.79	0.10
P_2_O_5_	0.00	0.00	8.32	0.89	2.38	0.31
K_2_O	0.00	0.00	8.32	0.89	2.38	0.31
Pesticides	3.42	0.50	3.23	0.34	1.36	0.17
Diesel	116.34	20.37	122.30	13.01	113.31	14.53
Water	197.27	29.60	194.43	20.68	113.39	14.54

## Results and discussion

Energy willow cultivation technologies were quite similar in all area groups. Preparation of the soil consisted of mechanical working, i.e. plowing, cultivating, and harrowing. Sometimes disking or tilling was also performed. The planting of the willow was done manually. Weeding was performed manually, or with tractor hoers. During the harvest, slat mowers were used only sporadically to cut willow, in the group of the larger plantations, while most often the harvesting was done manually, using pruning shears. The means of transport were used only partially during the harvesting as the harvested biomass was immediately loaded on trailers. The use of forestry harvesters could solve the problem to some degree as they could be relatively easily adjusted to biomass harvesting^74^. Transport of cut biomass and means of production, e.g. fertilizers or seedlings, was carried out using agricultural tractors and trailers. The low level of mechanization of the works resulted mainly from small areas of willow plantations, which is confirmed by research by Kwaśniewski^75^. He reports that in southern Poland the willow plantations are usually of small acreage, and scattered from each other, which is a certain economic barrier to the use of mechanized technologies.

Table 5 presents the results of the ReCiPe Midpoint analysis in individual area groups. Generally, it can be stated that as the area increased, the unit impact of processes decreased in all the analyzed categories. In the case of materials, in 13 out of 18 categories, an increase in the characterization factors was observed as the plantation area increased.

**Table 5:** Characterization factors of the ReCiPe Midpoint environmental analysis in area groups.

Midpoint indicators	Unit	Group I		Group II		Group III	
		Material	Processes	Material	Processes	Material	Processes
Climate change	kg CO2-eq	86.089	1344.508	141.465	1257.954	189.086	739.108
Ozone depletion	kg CFC-11-eq	0.00007	0.00023	0.00007	0.00021	0.00008	0.00012
Terrestrial acidification	kg SO2-eq	0.650	8.125	1.189	7.627	1.369	4.604
Freshwater eutrophication	kg P-eq	0.044	0.111	0.077	0.104	0.076	0.071
Marine eutrophication	kg N-eq	0.545	0.684	0.038	0.430	0.044	0.259
Human toxicity	kg 1,4-DB-eq	22.719	214.968	63.518	201.119	75.311	125.964
Photochemical oxidant formation	kg NMVOC	0.612	14.638	0.646	11.832	0.775	7.091
Particulate matter formation	kg PM10-eq	0.217	3.770	0.415	3.521	0.463	2.197
Terrestrial ecotoxicity	kg 1,4-DB-eq	0.504	0.486	0.028	0.130	0.027	0.070
Freshwater ecotoxicity	kg 1,4-DB-eq	6.539	90.343	2.381	33.798	2.902	17.509
Marine ecotoxicity	kg 1,4-DB-eq	0.909	36.541	2.177	30.367	2.684	15.799
Ionising radiation	kBq U235-eq	30.664	105.811	35.736	98.075	38.217	57.547
Agricultural land occupation	m2 yr	3.493	50.387	5.212	27.673	6.239	25.220
Urban land occupation	m2 yr	2.640	37.193	3.562	13.583	4.276	8.809
Natural land transformation	m2	0.359	9.585	0.134	0.421	0.147	0.243
Water depletion	m3	9.389	30.080	3.813	3.387	2.913	2.094
Metal depletion	kg Fe-eq	4.718	116.728	14.016	96.473	18.477	65.976
Fossil depletion	kg oil-e	132.107	465.504	143.241	426.961	153.978	248.762

Table 6 shows the results of the ReCiPe Midpoint analysis of the used production materials, as per crop area. Diesel fuel was consumed in all treatments, while mineral fertilizers, pesticides, and water were additionally used during fertilization and chemical protection treatments. Upon analyzing the environmental impact of the materials used, the decisive share of mineral fertilizers can be observed, although they were not heavily used due to the relatively good quality of the soil. Significant environmental impacts are also associated with the cultivation procedures and transportation. Although the only production material used in soil cultivation and transport was diesel fuel, the relatively high unit fuel consumption (in soil cultivation) and the high yield of willow biomass (in transport) result in a significantly high diesel fuel consumption and thus, a significant environmental impact.

**Table 6:** Characterization factors of the ReCiPe Midpoint environmental analysis for the used production materials (materials).

Midpoint indicators	Unit	Total	Soil preparat	Mineral fertilizat	Chemical care	Mechan. care	Harvest	Transport
Climate change	kg CO2-eq	138.880	27.291	66.140	24.549	1.941	2.728	16.231
Ozone depletion	kg CFC-11-eq	0.000	0.00003	0.00001	0.00001	0.00000	0.00000	0.00002
Terrestrial acidification	kg SO2-eq	1.070	0.239	0.500	0.148	0.017	0.024	0.142
Freshwater eutrophication	kg P-eq	0.066	0.003	0.036	0.025	0.000	0.000	0.002
Marine eutrophication	kg N-eq	7.209	2.600	0.017	2.998	0.289	0.001	1.306
Human toxicity	kg 1,4-DB-eq	53.849	4.172	36.695	9.787	0.297	0.417	2.481
Photochemical oxidant format	kg NMVOC	0.678	0.195	0.225	0.112	0.015	0.017	0.114
Particulate matter format	kg PM10-eq	0.365	0.070	0.185	0.057	0.005	0.007	0.041
Terrestrial ecotoxicity	kg 1,4-DB-eq	1.820	0.652	0.011	0.757	0.072	0.000	0.328
Freshwater ecotoxicity	kg 1,4-DB-eq	3.941	0.574	1.386	1.602	0.057	0.018	0.305
Marine ecotoxicity	kg 1,4-DB-eq	1.923	0.168	1.297	0.333	0.013	0.015	0.098
Ionising radiation	kBq U235-eq	34.872	12.608	7.121	5.489	0.897	1.260	7.498
Agricultural land occupation	m2 yr	4.981	0.499	2.952	1.173	0.042	0.034	0.282
Urban land occupation	m2 yr	3.493	0.417	2.113	0.662	0.034	0.030	0.237
Natural land transformation	m2	0.213	0.083	0.012	0.058	0.007	0.006	0.048
Water depletion	m3	5.372	1.496	1.501	1.412	0.154	0.030	0.779
Metal depletion	kg Fe-eq	12.404	0.914	9.121	1.680	0.068	0.084	0.537
Fossildepletion	kg oil-e	143.109	61.418	19.076	15.589	4.370	6.134	36.521

Table 7 shows the results of the ReCiPe Midpoint analysis of the used technical means of production, as per crop area. Among all impact categories, the processes related to the use of transport take by far the dominant position. As already mentioned, this is due to the high yield of energy willow biomass and the applied harvesting technology, which required using means of transport and significantly increased their working time. Murphy^7^ identifies transport as one of the three key processes in the production chain with the greatest environmental impact of all considered categories. Processes requiring the use of soil cultivation machinery rank second in terms of environmental impact, which results from the significant labor intensity of soil cultivation.

**Table 7:** Characterization factors of the ReCiPe Midpoint environmental analysis for the used technical means of production (processes).

Midpoint indicators	Unit	Total	Soil preparat	Mineral fertilizat	Chemical care	Mechan. care	Harvest	Transport
Climate change	kg CO2-eq	1113.857	209.420	4.849	10.275	9.822	5.850	873.641
Ozone depletion	kg CFC-11-eq	0.00019	0.00003	0.00000	0.00000	0.00000	0.00000	0.00015
Terrestrial acidification	kg SO2-eq	6.785	1.469	0.036	0.070	0.066	0.039	5.106
Freshwater eutrophication	kg P-eq	0.095	0.029	0.001	0.003	0.002	0.001	0.059
Marine eutrophication	kg N-eq	112.424	17.874	0.002	0.801	0.999	0.002	92.746
Human toxicity	kg 1,4-DB-eq	180.684	41.234	1.223	3.697	3.355	1.613	129.561
Photochemical oxidant format	kg NMVOC	11.187	2.340	0.055	0.108	0.102	0.056	8.528
Particulate matter format	kg PM10-eq	3.163	0.805	0.018	0.034	0.033	0.021	2.252
Terrestrial ecotoxicity	kg 1,4-DB-eq	28.162	4.466	0.000	0.201	0.250	0.000	23.245
Freshwater ecotoxicity	kg 1,4-DB-eq	47.217	5.364	0.049	0.437	0.498	0.064	40.803
Marine ecotoxicity	kg 1,4-DB-eq	27.569	1.927	0.046	0.152	0.161	0.061	25.222
Ionising radiation	kBq U235-eq	87.144	16.157	0.383	0.851	0.786	0.463	68.505
Agricultural land occupation	m2 yr	34.426	17.567	0.301	1.508	2.384	0.601	12.066
Urban land occupation	m2 yr	19.862	4.229	0.059	0.353	0.519	0.114	14.587
Natural land transformation	m2	3.416	0.267	0.001	0.016	0.020	0.002	3.110
Water depletion	m3	11.854	2.073	0.016	0.115	0.127	0.024	9.499
Metal depletion	kg Fe-eq	93.059	30.276	0.573	2.394	2.660	1.076	56.081
Fossil depletion	kg oil-e	380.409	68.757	1.608	3.225	3.039	1.847	301.933

Table 8 shows the results of the Midpoint environmental analysis of the fertilizers and pesticides only, as per crop area. Mineral fertilizer use is dominant in this impact category, although it was used less frequently than the pesticides (mainly herbicides, to control weeds). The highest amount of pesticides as per crop area was used in Group I, while the highest amount of fertilizers was used in Group II (Table 4). The use of fertilizers results in a climate change impact of 36,107 kg CO2-eq, while the use of pesticides results in half the amount of kg CO2-eq. According to Keoleian and Volk^14^, fertilizer use is responsible for 75% of GHG emissions caused by agricultural inputs associated with energy willow cultivation.

**Table 8:** Characterization factors of the ReCiPe Midpoint environmental analysis for the used fertilizers and pesticides.

Midpoint indicators	Unit	Mineral fertilizers	Pesticides
Climate change	kg CO2-eq	36.107	18.594
Ozone depletion	kg CFC-11-eq	0.000003	0.000004
Terrestrial acidification	kg SO2-eq	0.249	0.108
Freshwater eutrophication	kg P-eq	0.014	0.024
Marine eutrophication	kg N-eq	0.009	0.007
Human toxicity	kg 1,4-DB-eq	16.527	9.081
Photochemical oxidant format	kg NMVOC	0.107	0.065
Particulate matter format	kg PM10-eq	0.086	0.042
Terrestrial ecotoxicity	kg 1,4-DB-eq	0.005	0.008
Freshwater ecotoxicity	kg 1,4-DB-eq	0.628	0.320
Marine ecotoxicity	kg 1,4-DB-eq	0.589	0.294
Ionising radiation	kBq U235-eq	3.062	3.381
Agricultural land occupation	m2 yr	1.296	0.930
Urban land occupation	m2 yr	0.809	0.259
Natural land transformation	m2	0.006	0.004
Water depletion	m3	0.899	0.45917
Metal depletion	kg Fe-eq	4.189	1.370
Fossil depletion	kg oil-e	9.671	6.039

**Figure 3:**
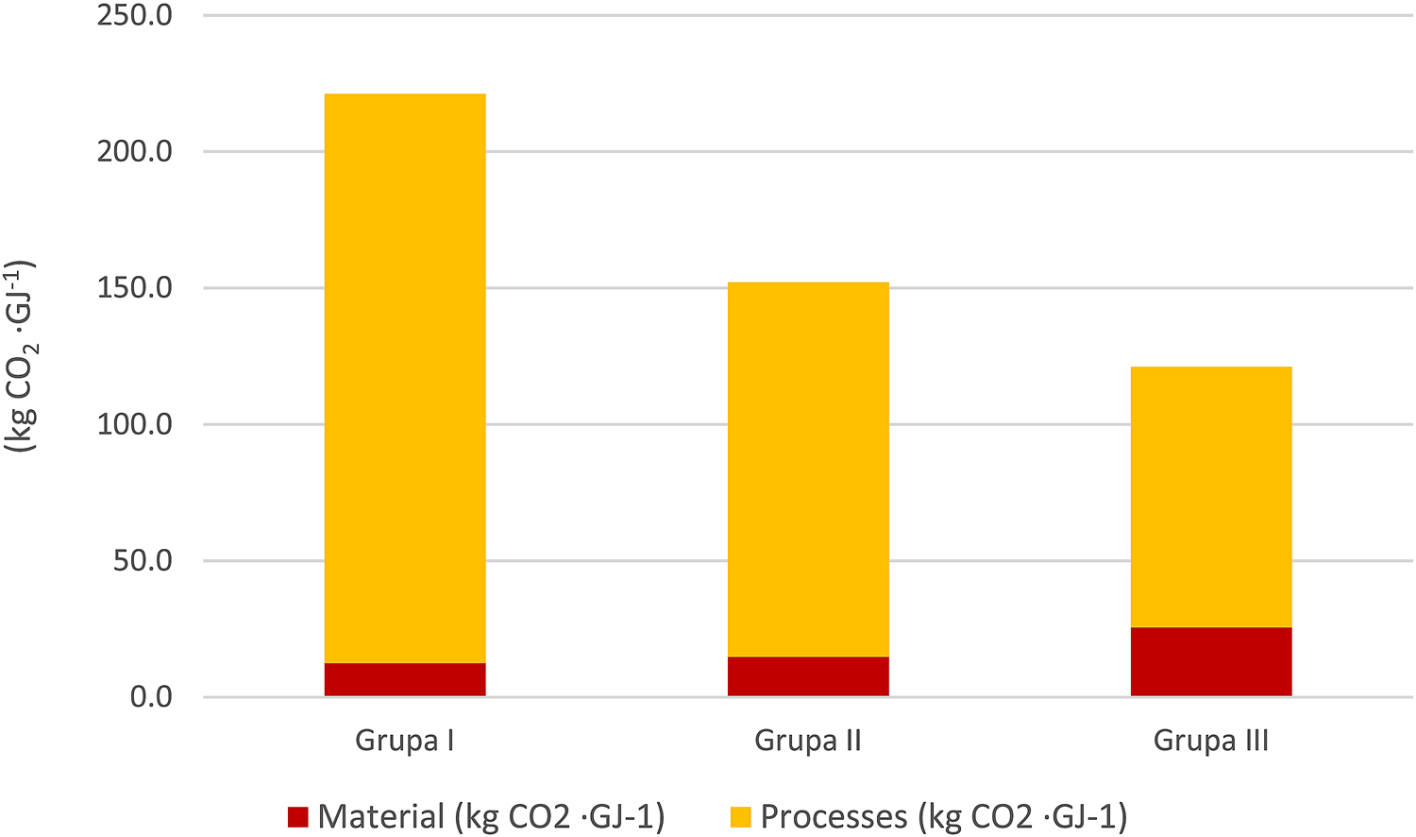
Structure of CO_2_ emissions in area groups.

Table 9 and Fig. 3 present the level of CO_2_ emissions in area groups calculated per the calorific value of fresh weight of the harvested willow. It can be observed that a decisively higher emission level, approx. 221 kg CO_2_· GJ^-1^, occurs in the smallest plantations, and in the group of the largest plantations, it decreases to 121 kg CO_2_· GJ^-1^. The decisive share in emissions (from 79 to 95%) belongs to processes.

**Table 9:** Structure of CO_2_ emissions in area groups (kg CO_2_·GJ^−1^).

Specification		Group I	Group II	Group III
Soil preparation	Material	4.53	2.55	3.43
	Processes	31.29	22.15	26.27
Mineral fertilization	Material	0.00	7.56	17.48
	Processes	0.00	0.57	1.26
Chemical care	Material	5.05	2.61	1.73
	Processes	1.35	1.36	1.08
Mechanical care	Material	0.55	0.14	0.12
	Processes	1.85	1.00	0.92
Harvesting	Material	0.00	0.00	1.03
	Processes	0.00	0.00	2.16
Transport	Material	2.33	2.01	1.88
	Processes	174.17	112.15	63.75

Energy willow cultivation doesn’t produce large CO_2_ emissions. Compared with the coal system, the combustion of just biomass pellets to generate 8,300 GWh of power can reduce global warming impacts by 7.9 million tons of CO_2_-eq, which is equivalent to an 85% reduction in GHG emissions, according to Wiloso et al.^76^. It should also be remembered that energy crops store CO_2_ in the soil and roots, which according to Yang and Tilman^77^ is a more important determinant in the climate change mitigation potential of biofuels than the above-ground biomass. According to Heller et al.^78^, power production from willow biomass is nearly GHG-neutral (40–50 kg CO_2_ eq./MWh of electric power produced).

Table 10 presents the environmental impact of the consumption of production materials used in the cultivation of energy willow as per the plantation area. The environmental impact of production materials was considered on three levels, namely: human health, ecosystems, and resources. In the case of chemical plant protection and mechanical weeding, the environmental impact associated with the use of production materials decreased as the group's area increased. This state of affairs results from a lower intensity of pesticide use per hectare of cultivation, which is confirmed by the data presented in Table 4, as well as from less frequent mechanical weeding operations on larger plantations (lower fuel consumption). Both chemical and mechanical procedures were not compensated in any way by the use of other methods of weed control in larger willow cultivation areas, which is reflected in the decrease in the fresh crop yield (Table 2). The environmental impact (EI) of material consumption is also quite unequivocal in the case of mineral fertilization, yet reversed: as the group area increases so does the EI, which is the result of increasing the unit consumption of fertilizers in the second group. In turn, in the group of the largest plantations, the doses of fertilizers per unit area were slightly lower than in Group II (Table 4), but higher power tractors were used for fertilization, which resulted in significantly higher fuel consumption. Plant life cycle research usually indicates fertilization as a treatment that generates the greatest environmental impact^79^. Other technological procedures are no longer so unequivocal in terms of environmental impact. The environmental impact associated with transportation is the highest in Group II and the lowest in Group III, which correlates with the transport distances shown in Table 1. In turn, the mechanical harvest was used only in the group of the largest plantations, hence the production materials (fuel) affected the environment in this group. Upon analyzing the structure of the environmental impact on three levels, i.e. human health, ecosystems, and resources (Table 10), it can be observed that for all technological procedures, except mineral fertilization, the structure is dominated by resources, which generally exceeds the value of other streams of environmental impact. Only in the case of mineral fertilization, the dominant impact is human health, which results from the properties of mineral fertilizers used. The level of environmental impact in the resources category ranges from 0.00 Pt for mineral fertilization in Group I and harvest in Groups I and II, to 7.54 Pt for soil preparation activities in Group I. The environmental impact level in terms of human health ranges from 0.00 Pt for mineral fertilization (Group I) and harvest (Groups I and II), to 5.94 Pt also for mineral fertilization in Group III. In turn, in the category of ecosystems, the magnitude of the environmental impact ranged between 0.00 Pt (mineral fertilization in Group I) to 2.60 Pt, also for activities related to mineral fertilization, but in Group III (Table 10).

**Table 10:** The environmental impact of the consumption of production material, as per plantation area (Pt·ha^−1^).

Group	Category	Soil preparation	Mineral fertilization	Chemical care	Mechanical care	Harvesting	Transport
I	Human Health	1.343	0.000	1.609	0.149	0.000	0.674
	Ecosystems	0.854	0.000	0.758	0.095	0.000	0.429
	Resources	7.545	0.000	2.091	0.838	0.000	3.789
II	Human Health	1.062	3.947	1.089	0.056	0.000	0.801
	Ecosystems	0.675	1.579	0.527	0.036	0.000	0.509
	Resources	5.965	3.449	1.768	0.317	0.000	4.499
III	Human Health	1.127	5.941	0.590	0.046	0.353	0.625
	Ecosystems	0.717	2.606	0.300	0.029	0.225	0.398
	Resources	6.334	3.948	1.319	0.256	1.984	3.514

Table 11 presents the environmental impact of applying technical means of production (processes) in the cultivation technologies of energy willow, as per the plantation area. The environmental impact of machines and devices was considered on three levels: human health, ecosystems, and resources, similar to the case of production materials. In the case of all technological activities except chemical plant protection, the environmental impact of the use of machines and devices in particular groups is shaped quite clearly. As the plantation area increases in groups, it decreases (soil preparation, mechanical treatment, transportation) or increases (mineral fertilization, harvest). In the case of chemical protection, mechanical cultivation, and transport—the environmental impact resulting from processes per hectare of plantation decreases as the area increases. This is primarily due to the number of procedures performed or the transport distance. In their research,Goglioa^80^ and Kowalczyk^57^ indicate the great importance of the distance of biomass transport in the aspect of environmental protection. When analyzing technological procedures related to mineral fertilization and harvesting, the lowest environmental impact of EI processes was recorded in the group of plantations with the smallest acreage, and the highest—in Group III. In Group I, no mineral fertilization was used, and the harvest was done manually, hence the lack of an environmental impact of the use of technical means of production. The lesser environmental impact of chemical plant protection in the largest plantation group, as compared to Group II, is due to the lesser number of chemical sprayings. Upon analyzing the structure of the environmental footprint related to processes (the use of machinery and equipment) on the said three levels, i.e. human health, ecosystems, and resources (Table 11), it can be observed that for all technological procedures, the structure is dominated by the human health category, which generally exceeds significantly the value of the other environmental impact streams. The level of the environmental impact of processes in the human health category ranges between 0.00 Pt for mineral fertilization (Group I) and harvest (Groups I and II), to 47.76 Pt for transportation in Group I. The impact level in terms of resources ranges from 0.00 Pt for mineral fertilization (Group I) and harvest (Groups I and II), to 43.93 Pt for transportation in Group I. In turn, the magnitude of environmental impact for the ecosystems category ranged between 0.00 Pt (mineral fertilization category in Group I) and harvest (Groups I and II) to 21.81 Pt for transportation in Group I.

**Table 11:** The environmental impact of the utilization of technical means of production (processes) as per plantation area (Pt·ha^−1^).

Group	Category	Soil preparation	Mineral fertilization	Chemical care	Mechanical care	Harvesting	Transport
I	Human Health	13.833	0.000	0.631	0.894	0.000	47.763
	Ecosystems	6.023	0.000	0.302	0.482	0.000	21.812
	Resources	11.444	0.000	0.554	0.784	0.000	43.931
II	Human Health	13.599	0.273	0.758	0.477	0.000	43.933
	Ecosystems	5.840	0.115	0.363	0.257	0.000	20.063
	Resources	11.139	0.224	0.665	0.418	0.000	40.408
III	Human Health	10.296	0.455	0.421	0.397	0.654	21.133
	Ecosystems	4.441	0.192	0.201	0.214	0.295	9.651
	Resources	8.442	0.373	0.369	0.349	0.557	19.437

Table 12 presents the environmental impact of the consumption of production materials used in energy willow cultivation technologies, as per fresh biomass yield. As in the previous tables, the analysis of the environmental impact was carried out in three categories: human health, ecosystem, and resources. The environmental impact of material consumption (per biomass yield) for individual technological procedures is almost identical as it is for the crop area unit (Table 4). Only in the case of transportation works, the environmental impact decreases very slightly along with the increase of plantation area in the group. Similarly to Table 11, the structure of the environmental impact was also determined in the following categories: human health, ecosystems, and resources. Due to a different reference unit (fresh willow biomass yield), the level of environmental impact in the resources category ranges from 0.00 Pt for mineral fertilization in Group I and harvest (Groups I and II), to 1.09 Pt for soil preparation activities in Group I. The magnitude of environmental impact in the category of human health ranges from 0.00 Pt for mineral fertilization (Group I) and harvest (Groups I and II), to 0.82 Pt also for mineral fertilization in Group III. In turn, in the category of ecosystems, the level of environmental impact ranged from 0.00 Pt (mineral fertilization in Group I) to 2.60 Pt, for activities related to mineral fertilization, but in Group III.

**Table 12:** The environmental impact of the consumption of production materials as per biomass yield (Pt·tonne^−1^).

Group	Category	Soil preparation	Mineral fertilization	Chemical care	Mechanical care	Harvesting	Transport
I	Human Health	0.195	0.000	0.226	0.024	0.000	0.100
	Ecosystems	0.124	0.000	0.107	0.015	0.000	0.064
	Resources	1.098	0.000	0.294	0.133	0.000	0.564
II	Human Health	0.110	0.411	0.116	0.006	0.000	0.087
	Ecosystems	0.070	0.165	0.056	0.004	0.000	0.055
	Resources	0.618	0.359	0.188	0.033	0.000	0.487
III	Human Health	0.148	0.825	0.077	0.005	0.044	0.081
	Ecosystems	0.094	0.362	0.039	0.003	0.028	0.052
	Resources	0.831	0.548	0.174	0.029	0.249	0.457

The environmental impact of the use of technical means of production (processes) in energy willow cultivation technologies, as per the fresh willow biomass yield, was presented in Table 13. Similarly to production materials, the environmental impact of processes was considered in three categories: human health, ecosystems, and resources. The dependence of the environmental impact on the use of machines and devices in individual plantation groups differs but slightly from the results analyzed in Table 11, the only exception being the chemical plant protection treatments. The structure of the environmental impact associated with processes (the use of machines and devices) on three levels: human health, ecosystems, and resources, is as in Table 11. The level of the environmental impact of processes in the category of human health ranges from 0.00 Pt for mineral fertilization (Group I) and harvest (Groups I and II), to 7.49 Pt for transportation in Group I. The level of environmental impact in the category of resources ranges from 0.00 Pt for mineral fertilization (Group I) and harvest (Groups I and II), to 6.89 Pt for transportation in Group I. In turn, in the category of ecosystems, the magnitude of the environmental impact ranged between 0.00 Pt (mineral fertilization category in Group I) and harvest (Groups I and II) to 3.42 Pt for transportation in Group I.

**Table 13:** The environmental impact of the utilization of technical means of production (processes) as per biomass yield (Pt·tonne^−1^).

Group	Category	Soil preparation	Mineral fertilization	Chemical care	Mechanical care	Harvesting	Transport
I	Human Health	2.017	0.000	0.089	0.143	0.000	7.498
	Ecosystems	0.879	0.000	0.043	0.077	0.000	3.424
	Resources	1.668	0.000	0.078	0.125	0.000	6.896
II	Human Health	1.424	0.028	0.081	0.050	0.000	4.828
	Ecosystems	0.612	0.012	0.039	0.027	0.000	2.205
	Resources	1.167	0.023	0.071	0.044	0.000	4.441
III	Human Health	1.319	0.063	0.053	0.046	0.079	2.745
	Ecosystems	0.569	0.027	0.026	0.025	0.036	1.253
	Resources	1.081	0.052	0.047	0.040	0.067	2.524

**Figure 4:**
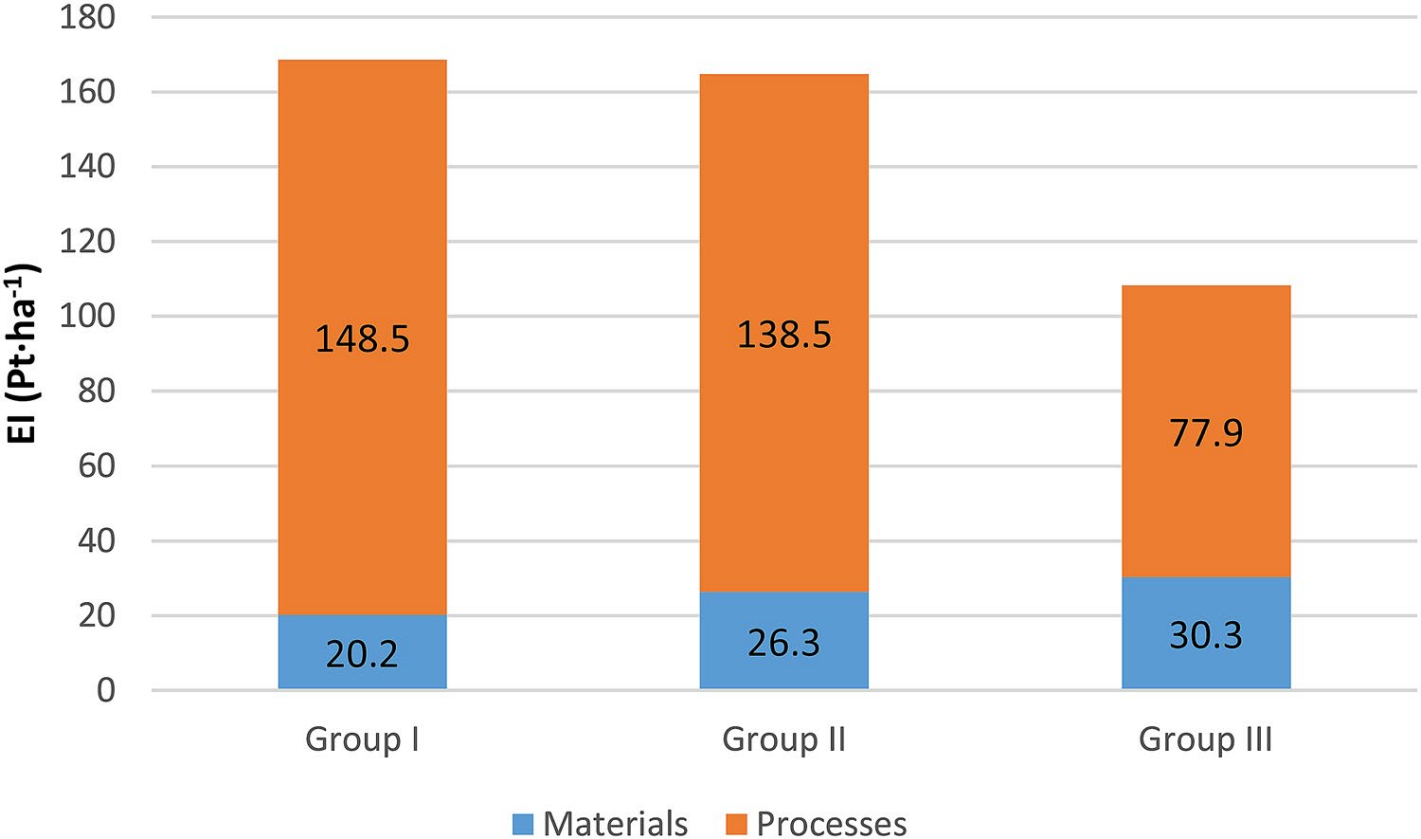
The structure of the total environmental impact as per plantation area.

Table 14 and Fig. 4 present the unit total environmental impact (as per plantation area) related to the energy willow cultivation technology, broken down into the impact of materials and processes. The total environmental impact in Groups I and II is very similar and amounts to 168 Pt and 164 Pt. The total environmental impact in the group of the largest plantations is the most favorable and amounts to only 108 Pt. To compare, the environmental impact in potato cultivation, estimated using the same methodology, is approx. 280 Pt^48^. Upon analyzing the results in Fig. 4, one observes a much greater environmental impact of processes, i.e. the use of machinery and tools, than of production materials. The processes in Group I affect 88% of the total environmental impact; in Group II it is 84%, and in the group of the largest plantations—72%. Materials, in turn, decide on the level of the total environmental impact, from 12% in Group I, to 16% in Group II, to 28% in the group of plantations with the largest area. Thus, a clear tendency of the impact of processes/materials on the overall environmental impact to increase/decrease can be observed along with the increase in the plantation area.

**Table 14:** The structure of the total environmental impact as per plantation area (Pt·ha^−1^).

Specification		Group I	Group II	Group III
Soil preparation	Material	9.74	7.70	8.18
	Processes	31.30	30.58	23.18
Mineral fertilization	Material	0.00	8.98	12.50
	Processes	0.00	0.61	1.02
Chemical care	Material	4.46	3.38	2.21
	Processes	1.49	1.78	0.99
Mechanical care	Material	1.08	0.41	0.33
	Processes	2.16	1.15	0.96
Harvesting	Material	0.00	0.00	2.56
	Processes	0.00	0.00	1.51
Transport	Material	4.89	5.81	4.54
	Processes	113.51	104.40	50.22

**Figure 5:**
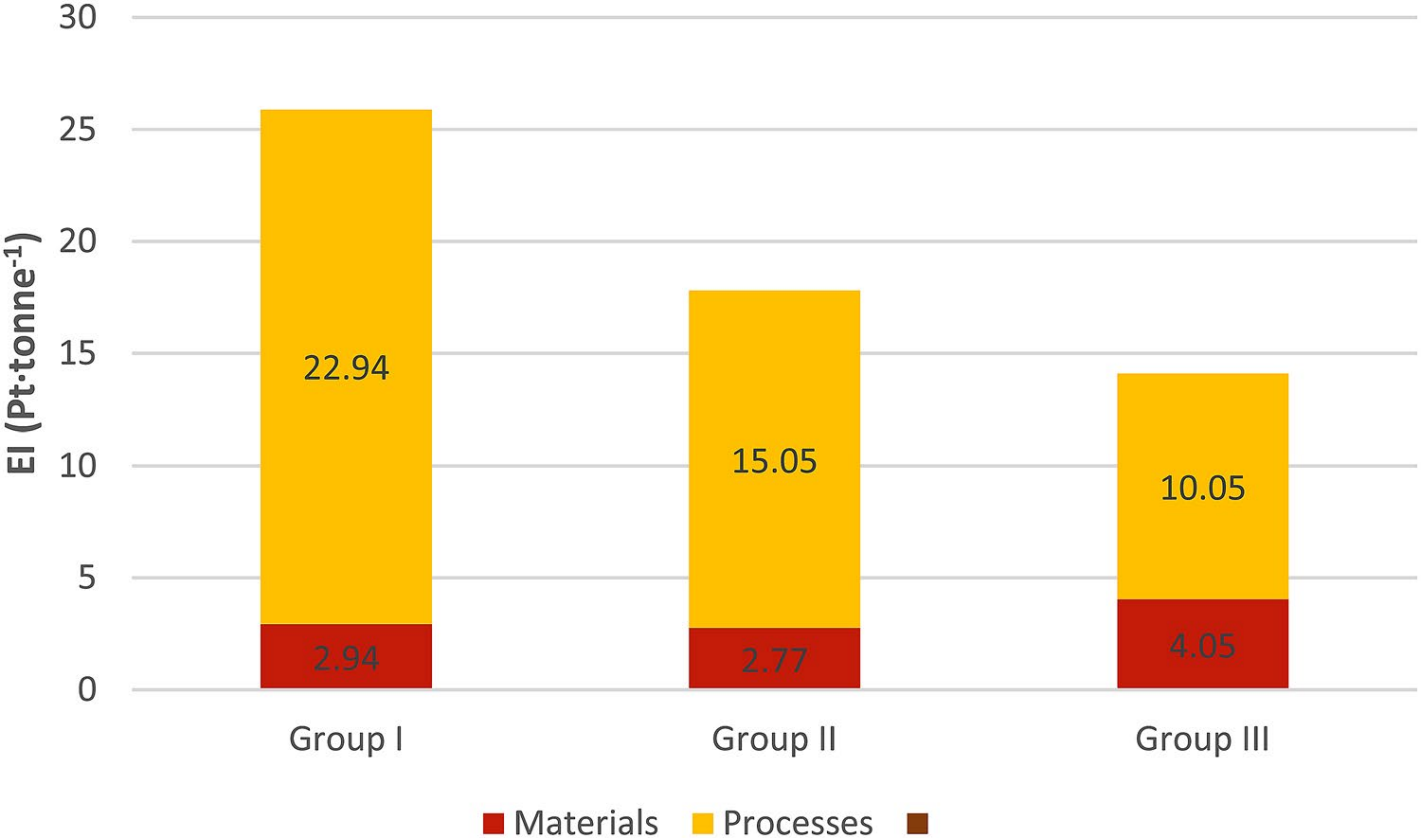
The structure of the total environmental impact as per the biomass yield.

Table 15 and Fig. 5 show the structure of the unit total environmental impact (as per fresh biomass yield) related to the energy willow cultivation technology in individual area groups. This structure, similar to Fig. 4, relates to the interaction of materials and processes. The total environmental impact is at a completely different level than that observed in Fig. 4 (as per plantation area) and is calculated per yield of fresh willow biomass, from 14 Pt in the smallest plantation group to 18 Pt in the middle group and 26 Pt in the largest plantation group. Undoubtedly, the shaping of the above values is affected by biomass yields in individual groups. The results of the Endpoint analysis for particular area groups are consistent with the results of the Midpoint analysis, i.e. a lower environmental impact, both for the growing area and the biomass yield is observed in the cultivation of energy willow on large plantations. This state of affairs results, among other things, from better organization of work, especially related to the use of means of transport, which is evidenced by the results in Tables 14 and 15.

**Table 15:** The structure of the total environmental impact as per biomass yield (Pt·tonne^−1^).

Specification		Group I	Group II	Group III
Soil preparation	Material	1.42	0.80	1.07
	Processes	4.56	3.20	2.97
Mineral fertilization	Material	0.00	0.94	1.74
	Processes	0.00	0.06	0.14
Chemical care	Material	0.63	0.36	0.29
	Processes	0.21	0.19	0.13
Mechanical care	Material	0.17	0.04	0.04
	Processes	0.35	0.12	0.11
Harvesting	Material	0.00	0.00	0.32
	Processes	0.00	0.00	0.18
Transport	Material	0.73	0.63	0.59
	Processes	17.82	11.47	6.52

## Conclusions and the further research

Based on the conducted research, it was found that:

1. A decisively dominant position in the structure of the environmental impact (EI), related to the cultivation of energy willow, is held by the so-called processes, i.e. the use of technical means of production. Their share in the total environmental impact decreases from 148.5 Pt in the group of the smallest plantations, to 77.9 Pt in the group of the largest plantations. This state of affairs should prompt energy willow biomass producers to simplify the cultivation and reduce the number of technological treatments applied.
2. The cultivation of energetic willow on larger plantations is characterized by a much lower environmental impact (as per the cultivation area), at approx. 108 Pt, compared to the cultivation on smaller plantations, where the value of the environmental impact is 168 Pt. This is due to, e.g. a certain simplification of production technology, as well as a reduction in the use of production materials (fertilizers, pesticides). Unfortunately, these adversely affect the volume of the harvested biomass.
3. In the structure of the environmental impact of production materials used in the cultivation of energy willow, the dominant position (from 57% in Group III to 70% in Group I) is held by the category of resources. Its size depends largely on the level of diesel fuel consumption, i.e. the number of harvest runs and the technical equipment’s level of advancement.
4. In the case of the environmental impact of processes (the use of technical means of production), the largest percentage, approx. 42%, was observed in the human health category, which is also mainly associated with the consumption of fuel, i.e. diesel.
5. The unfavorable impact of production materials applies to the greatest extent to soil preparation and slightly less to mineral fertilization, which once again points to diesel oil as a means of production that is particularly important in the aspect of environmental protection. It is also an argument for the expediency of seeking alternative fuels, the use of which will significantly contribute to environmental protection.
6. Further study of energy willow in subsequent years of cultivation is necessary to better understand its environmental impact and to balance the possible environmental benefits of producing various types of energy and heat from biomass. Further research will include a life cycle analysis on various willow biomass conversion technologies. An economic analysis of biofuel production in terms of the environmental impact of production will also be conducted.
